# The Use of Electricity in Acne Vulgaris and Acne Rosacea1Read at the eighth annual meeting of the American Electro-Therapeutic Association, at Buffalo, September 13-15. 1898.

**Published:** 1898-11

**Authors:** Grover William Wende

**Affiliations:** Buffalo, N. Y., Clinical professor of skin diseases, University of Buffalo


### THE USE OF ELECTRICITY IN ACNE VULGARIS AND ACNE ROSACEA.[1]

By GROVER WILLIAM WENDE, M. D., Buffalo, N. Y.,
Clinical professor of skin diseases, University of Buffalo.

NOWHERE within the wide domain of medical science can be found a class of diseases so manifold in manifestations, so embarrassing to the patient and so trying to the physician, as the class known as "the acnes." These consist of two distinct affections—namely, acne vulgaris and acne rosacea. Each of them possesses peculiar and striking features, the one being marked by its well-defined varieties and the other by its characteristic stages and complications.

Acne vulgaris consists of an inflammatory and functional disorder of the skin, having its seat in and around the sebaceous glands; while acne rosacea is an abnormal chronic hyperemia of the face, characterised by redness, dilatation, enlargement of the blood-vessels and connective tissue hypertrophy. It usually consists of two processes, an acne and a rosacea, the acne being secondary.

The discussion of these maladies, generally so insignificant and harmless, might, at first glance, appear quite common-place were it not for their frequent occurrence and chronic course. However, an honest practitioner, estranged by a groundless suspicion of a blood dyscrasia, will, upon careful investigation, establish many interesting points of practical value regarding their true nature and therapy—points not so readily discernible in other skin affections—which will well reward his efforts to bring about a cure.

The fundamental idea of the treatment of acne is to remove disfigurements by the employment of such local applications to the

---

1. Read at the eighth annual meeting of the American Electro-Therapeutic Association, at Buffalo, September 13-15, 1898.

affected parts as are calculated to produce stimulation and discourage hyperemia. The attempt is then made to remove the cause of the disease by internal administrations. Here we are often at a loss, by reason of the various concealed contingents of the affection. Hence the treatment of acne, as a rule, depends upon two factors, the local and the constitutional. The effort should always be made to exterminate the one from which the more danger is likely to result. We must endeavor to determine the source of the characteristic irritation before we can treat the disease intelligently and with any hope of success—in fact the only hope of successful treatment lies in an accurate knowledge of its etiology. Accordingly in many cases where the cause is definitely known the result is favorable; while, on the other hand, if the etiological factor is obscure, all remedies, no matter how much extolled, will utterly fail. The treatment of these conditions must depend on the special features of each case.

According to Dr. Bulkley, the causes of acne are: (1) General—pertaining to classes of cases; (2) Special—constitutional and systemic; (3) Local.

There are, however, numerous cases whose causes are ill-defined and which do not respond to ordinary treatment, but present features which demand special remedies. According to my experience, these should be assigned to the last division of the classification made by John Hunter and based exteriorly upon therapeutic results:

(1) Those that sulphur will cure; (2) Those that mercury will cure; (3) Those that the devil himself cannot cure.

Owing to the rapid strides made by electricity, cases of acne that were formerly found under this last heading only, have gradually grown less, so that, now, those that do not yield to ordinary treatment can, in a majority of cases, be successfully overcome by the use of the electric current. When this is applied to the cutaneous surface the process is characterised as electro-dermato-therapy. A large proportion of skin diseases is due either to an exalted or depressed condition of the nerves which govern circulation, excretion, secretion and nutrition, and the employment of electricity in these instances has been fully sanctioned by modern investigations.

It is not impossible that during the development of the body—from fifteen to twenty-five years—the excretory powers of the sebaceous glands are largely due to the influence of the nervous system. By reason of this, their functional activity is much increased and a greater amount of sebaceous matter is produced, whereby the common ducts and orifices are likely to become obstructed. It is indisputable that the more the subject is studied, the more certain it is to appear that an exalted or depressed condition of the nerves controls the secretions of the sebaceous glands. This is most apt to occur at puberty. Indirectly, the functional disturbance is frequently brought about by the influence of certain particular organs, in which case special consideration and treatment will be demanded.

In many cases the local cause of acne exists independently and will be found to be due to the sluggishness of the integument. The cutaneous muscles are deficient in tone and do not respond to the contractile power of the nerves controlling them, while the epidermis is thick, opaque and devoid of expression. This is the most favorable condition for the development of a general acne. These special conditions may be remedied by either general or special treatment.

Before adverting to the benefits to be derived from the application of electricity in certain forms of acne, it is important to note that the current may be employed to advantage in two different ways: (1) By electric stimulation and irritation, to be applied by means of the ordinary sponge-electrodes; (2) By electrolysis, for the destruction of chronic and indurated lesions, using the ordinary electric needle.

In the employment of the sponges it is not necessary or expedient to open the pustules or remove the comedones. Solution of continuity should be avoided. The galvanic current should be applied in exact accordance with the bipolar method, as follows: The electrodes, after having their surface properly covered with moist absorbent cotton, are placed upon the face, in close proximity to each other, and their position is constantly changed until the skin becomes well reddened. The current must be continuous and is to be regulated by the ability of the patient to endure pain. Should the skin be extremely sensitive, the anode can be held *in situ* and the cathode employed is gently stroking the face. To be thoroughly effective, five or ten cells are generally required; an application fifteen minutes in duration is usually sufficient to accomplish the desired result.

To cause a more sudden resolution in chronic manifestations, such as indurated papules, electrolysis is made use of, by puncturing the lesions at the center with an irido-platinum needle, as many times as the peculiar condition may seem to require. The puncture must be made by the galvano-negative; a current of from one to four milliampères will usually be sufficient.

My own experience with electricity in its therapeutical relations to acne vulgaris has been limited to twenty-two cases which have failed to respond to the usual remedies and methods. After a three months' trial, the application having been made on alternate days, three of the cases seemed to have received no benefit, eight were partially cured, while the remaining ones manifested but a temporary improvement. As in other forms of treatment, relapses occurred—a frequent sequence of acne; however, these cases would afterward respond to the current, showing the best results in the treatment of this particular condition by electricity.

The report of a case that had resisted every known treatment, but, at my hands, easily yielded to the forces of electricity, is here given to illustrate how intrinsically interesting and wonderful are the effects of this more modern agent in connection with acne, even in the most obstinate and defiant cases:

Josephine J., aged 35; first seen by me in June, 1895; family history unimportant; had suffered from acne since the age of 16. She presented no physical signs of disease; there were no digestive disturbances and no irregularities in menstruation. When she came to me, her cheeks, chin and forehead were literally covered with papules and pustules in different stages of formation; there were also numerous well-defined scars of a moderate character, one-eighth of an inch in diameter and especially noticeable upon the temples, neck and the upper and outer portions of the cheeks. The appearance of the patient would seem to indicate that she was suffering from a large papular syphilide; indeed, she had been treated for this affection on several occasions by other physicians. For two years she received from me the recognised treatment for the malady. Her diet was restricted and moderate, and increasing exercise was advised. The remedies which were administered internally mainly consisted of different alkalies, ergotine, iron and general tonics, as the exigencies of the case required. Externally, various applications, composed of well-known drugs, such as sulphur, bichloride of mercury, ichthyol, boracic acid and the like, in different proportions and combinations, were employed. Notwithstanding these efforts, and the benefit usually derived from these remedies in like cases, the lesions continued to develop. Eventually, it was determined to discard everything in the line of material agencies and to begin the use of electricity. The active and most prominent lesions, such as the large papules and pustules, were first destroyed by inserting into the center of each a sharp-pointed irido-platinum needle, connected with the negative pole, while the positive sponge-electrode, well moistened, was placed in the patient's hand. A weak current not exceeding five milliampères was employed and its effects were carefully watched. When the skin became blanched and a clear, white ring of froth formed around the needle it was withdrawn. In the majority of instances, two or three sittings were necessary to insure complete removal. Having thus cleared the face of the larger papules and pustules, the next thing was to remove the smaller lesions and erythematous blotches. This was accomplished by allowing the galvanic current to pass through moistened sponge-electrodes, placed in close proximity upon the face, and gently moved about until a sensation of warmth was felt by the patient and the skin was generally reddened. Usually it would be from fifteen to twenty-five minutes before the electrodes were removed. The applications were made on alternate days and extended over three months, when the patient was discharged as cured. The use of the needle left no scars. More than eight months have elapsed since the treatment was discontinued and there has been no return of the affection.

In considering acne rosacea, a few of the special influences at work in connection with the disease should be mentioned, that a rational idea may be formed concerning its proper treatment. The affection at first manifests itself as an angioneurosis, undoubtedly due to excessive irritability of the muscle-tone of the cutaneous vessels, characterised in the initial stage by prolonged or rapidly repeated stasis in the capillaries. All the superficial pathological changes are based upon this simple fact. It is interesting to observe in these cases the intimate connection of the sympathetic and the circulatory systems; this being especially marked in cases where there is rapid development and repeated change. When the indications seem to demand quickened circulation, for the purpose of aiding absorption and changing nutrition—these being usually apparent in the earlier stages of acne rosacea—we have every reason to believe, from analogy, that external galvanism will sooner restore the skin to its normal condition than the stimulating effect of sulphur or that of any other drug, topically applied. In addition to its effect in altering the local condition, it relieves the reflexes to which the disease is frequently due. We have thus a rational explanation of the origin and nature of the disease, for no part of the body can be healthy unless there is an harmonious interdependence between the histolytic and the histogenetic functions.

As an illustration of the beneficent results derived from the more direct electrical treatment by galvanism, the following case may be cited:

Mrs. K., aged 36; delicate and nervous constitution; married; has had two children; habitually constipated; poor circulation; hands and feet apt to be cold; subject to headache; very nervous immediately prior to menstruation; first affected six years ago. The face, on the cheeks, nose and forehead, on the mesial line, had become intensely red; the capillary vessels were dilated and a number of disseminated inflammatory papules were visible. The measures employed for this particular form of the affection were both local applications and constitutional drugs. No factor or feature of the case was overlooked; the dietary was modified, constipation was relieved and the uterine disorders were duly considered. There was, however, no improvement in the hyperemic nature of the difficulty and the nervous condition of the patient seemed to be augmented. But, however, after a time corresponding to that covering the regulation treatment, some eight or nine months ago, galvanism was employed by placing the anode on the abdomen over the solar plexus and gently stroking the affected parts of the face with the cathode. At the expiration of eight weeks the condition of the patient was materially ameliorated, clearly demonstrating the value of the electric method over all others.

In cases where hypertrophic changes ensue, with an actual increase of connective tissue, and the bloodvessels are tortuous and largely dilated, electrolysis must be resorted to, in addition to galvanism topically applied. It has been my own custom in applying the electric current to follow the plan adopted by Hardaway, which he claims is less painful and more effectual than to pass the same amount of current through the ordinary sponge-electrode. His plan consists in attaching the needle, or needles, used for the destruction of the hypertrophy and dilated blood-vessels to the anode, and in connecting the cathode with a bowl of water into which the patient introduces one finger or more, as may be necessary to complete the circuit and accomplish the desired result. In cases having a moderate amount of diffused redness, transversed by tortuous capillaries, a disk electrode should be used, in which may be placed ten or fifteen cambric needles for introduction into the affected skin. A weak current, measuring two or three milliampères, of one or two minutes' duration, is usually sufficient. The next insertion should include the edges of the places previously treated. The vigor of the application naturally varies and should be governed by its reaction, which is manifested by redness and tumefaction in the parts treated. If carefully watched, no inconvenience to the patient will result and the action of the current will be thoroughly satisfactory. No more than three or four areas should be treated at one sitting, nor should the same locality receive treatment oftener than once in a fortnight.

For the destruction of enlarged veins, a broad-pointed needle is recommended, which should be thrust into the vessel and a current of about three milliampères allowed to pass into the affected part. If the vein is long and tortuous, the operation must be repeated according to circumstances. A given portion of the surface can be successfully treated at one and the same time. If ordinary care is taken in the course of the operation, and it is skilfully performed, there will be no evidence of cicatrisation, even under a magnifying-glass.

In conclusion, we must all admire the use of electricity in the treatment of the disease here named. It is not only most beneficent to the public, but it is an exceedingly valuable remedy in the hands of the profession—more valuable than any other which is calculated to relieve rosacea employed either in medicine or surgery. It is especially efficient in connection with the peculiar appearance to which the terms "red light" and "grog-blossom" have so pertinently been applied by almost everybody, both laymen and professionals, and of which a classical instance is furnished in Shakespeare's wonderful description of the face of the doughty Bardolph:

> "His face is all bubukles and whelks and knobs and flames o' fire; and his lips blow at his nose, and it is like a coal of fire, sometimes plue and sometimes red."

471 DELAWARE AVENUE.

